# Low genomic diversity of *Legionella pneumophila* within clinical specimens

**DOI:** 10.1016/j.cmi.2018.03.004

**Published:** 2018-09

**Authors:** S. David, M. Mentasti, J. Parkhill, V.J. Chalker

**Affiliations:** 1) Centre for Genomic Pathogen Surveillance, Wellcome Genome Campus, Hinxton, Cambridge, CB10 1SA, United Kingdom; 2) Respiratory and Vaccine Preventable Bacteria Reference Unit, Public Health England, Colindale, NW9 5EQ, United Kingdom; 3) Pathogen Genomics, Wellcome Sanger Institute, Wellcome Genome Campus, Hinxton, Cambridge, CB10 1SA, United Kingdom

**Keywords:** Genomic diversity, *Legionella pneumophila*, Legionellosis, Legionnaires' disease, SBT, WGS

## Abstract

**Objectives:**

*Legionella pneumophila* is the leading cause of Legionnaires' disease, a severe form of pneumonia acquired from environmental sources. Investigations of both sporadic cases and outbreaks rely mostly on analysis of a single to a few colony pick(s) isolated from each patient. However, because of the lack of data describing diversity within single patients, the optimal number of picks is unknown. Here, we investigated diversity within individual patients using sequence-based typing (SBT) and whole-genome sequencing (WGS).

**Methods:**

Ten isolates of *L. pneumophila* were obtained from each of ten epidemiologically unrelated patients. SBT and WGS were undertaken, and single-nucleotide polymorphisms (SNPs) were identified between isolates from the same patient.

**Results:**

The same sequence type (ST) was obtained for each set of ten isolates. Using genomic analysis, zero SNPs were identified between isolates from seven patients, a maximum of one SNP was found between isolates from two patients, and a maximum of two SNPs was found amongst isolates from one patient. Assuming that the full within-host diversity has been captured with ten isolates, statistical analyses showed that, on average, analysis of one isolate would yield a 70% chance of capturing all observed genotypes, and seven isolates would yield a 90% chance.

**Conclusions:**

SBT and WGS analyses of multiple colony picks obtained from ten patients showed no, or very low, within-host genomic diversity in *L. pneumophila*, suggesting that analysis of one colony pick per patient will often be sufficient to obtain reliable typing data to aid investigation of cases of Legionnaires' disease.

## Introduction

*Legionella pneumophila* is a Gram-negative bacterium found in fresh-water and soil environments [1]. Human infection with *L. pneumophila* can cause legionellosis, which ranges from a mild, flu-like illness (Pontiac fever) to a severe and potentially fatal pneumonia (Legionnaires' disease). The usual route of infection is via inhalation of aerosols from a contaminated environmental source [2]. Commonly implicated sources include cooling towers, spa pools, decorative fountains, and water systems of large buildings.

When Legionnaires' disease cases occur, clinical isolates are usually characterized together with epidemiologically linked environmental isolates to help determine the source of the infection. To date, most clinical microbiological laboratories have relied on analysing a single clinical isolate, or a small number of clinical isolates, from each patient. However, existence of within-host diversity of *L. pneumophila*, which has been poorly studied, would have important implications for the interpretation of molecular typing data. Here, we used sequence-based typing (SBT) [3], [4] together with whole-genome sequencing (WGS) to investigate the diversity amongst multiple colony picks recovered from individuals.

## Methods

Ten colony picks were obtained [5] from single sputum samples of ten epidemiologically unrelated patients with sporadic Legionnaires' disease in England (Table 1). Isolates were stored at –80°C. DNA was extracted after 48–72 hours of incubation on buffered charcoal yeast-extract agar at 37°C using the Wizard kit (Promega UK, Southampton, UK), eluted in 1 x Tris-EDTA buffer (pH 8.0), and quantified using GloMax (Promega, UK). SBT was undertaken as described previously [3], [4]. WGS was performed on Illumina X10 with 150-bp paired-end reads. Raw data were submitted to the European Nucleotide Archive (study accession number PRJEB12239/ERP013693). Individual accession numbers are provided in Table 1.

**Table 1:** Number and details of single-nucleotide polymorphisms (SNPs) identified amongst ten isolates recovered from each of ten Legionnaires' disease patients

Patient	Age	Sex	Isolation date (and number of days post infection onset that sample was obtained)	Clinical information	Epidemiological information	ST	Reference genome (and size in bp)	Length of reference genome mapped (bp)	Range, mean and median number of SNPs between pairs	SNP locations and gene	Accession numbers
1	79	M	June 2015 (5)	Admitted to ITU	Nosocomial case	42	EUL 120 (3,430,562) [6]	3,376,590–3,378,610	0 SNPs between all		ERR1608296–ERR1608305
2	55	M	May 2015 (8)	Not provided	Travel-associated	42	EUL 120 (3,430,562) [6]	3,391,462–3,393,086	Range 0–2, mean 0.4, median 0	470,661 (*puuB*), 2,941,541 (*hemC*)	ERR1608306–ERR1608315
3	55	M	June 2016 (5)	Severe community-acquired pneumonia, diarrhoea, febrile	Travel-associated (Caribbean and Spain)	42	EUL 120 (3,430,562) [6]	3,296,732–3,298,909	0 SNPs between all		ERR2216341–ERR2216350
4	71	M	April 2016 (6)	Admitted to ITU	Travel-associated (Italy)	23	EUL 28 (3,509,586) [6]	3,369,571–3,371,148	0 SNPs between all		ERR1608316-ERR1608325
5	64	M	June 2016 (3)	Respiratory failure	Community-acquired	1	Paris (3,635,495) [10]	3,436,622–3,438,632	Range 0–1, mean 0.2, median 0	418,832 (*lpp0256*)	ERR2216351–ERR2216360
6	66	F	September 2016 (8)	Admitted to ITU	Travel-associated (Greece)	37	EUL 165 (3,474,638) [6]	3,332,884–3,334,163	0 SNPs between all		ERR2216391–ERR2216400
7	69	M	July 2016 (5)	Admitted to ITU, pneumonia, sepsis	Travel-associated	20	*De novo* assembly (3,560,463)	3,544,455–3,545,244	0 SNPs between all		ERR2216371–ERR2216380
8	69	M	August 2016 (2)	Admitted to ITU, sepsis	Community-acquired	477	*De novo* assembly (3,307,866)	3,291,099–3,292,328	Range 0–1, mean 0.2, median 0	2,402,083 (intergenic)	ERR2216381–ERR2216390
9	75	M	September 2016 (7)	Not provided	Travel-associated	2287	*De novo* assembly (3,344,279)	3,328,536–3,329,783	0 SNPs between all		ERR2216401–ERR2216410
10	46	M	July 2016 (15)	Not provided	Community-acquired	1522	*De novo* assembly (3,621,967)	3,589,885–3,591,537)	0 SNPs between all		ERR2216361–ERR2216370

ST, sequence type; bp, base pair; ITU, intensive therapy unit.

De novo assemblies were generated [6], and MLSTcheck was used to confirm the sequence type (ST) from them [7]—in particular ensuring that at least one of the *mompS* alleles matched that called by traditional SBT (since this gene is duplicated). Assemblies were annotated using Prokka v1.11 [8].

Single-nucleotide polymorphisms (SNPs) were called for each isolate by mapping to a reference genome of the same ST using the Burrows–Wheeler aligner [9]. Available reference genomes included ST1 (Paris), ST23 (EUL 28), ST37 (EUL 165) and ST42 (EUL 120) [6], [10]. For the rest (ST20, ST477, ST1522 and ST2287), de novo assemblies were used. A pipeline comprising SAMtools, mpileup and BCFtools was used to call SNPs [11]. Various filters ensured high-accuracy base-calling, and bases were uncalled if any criteria were not fulfilled (reads matching base ≥4, reads matching base per strand ≥2, ratio of first to second base call ≥0.75, variant quality ≥50, mapping quality ≥30, strand bias ≥0.001, map bias ≥0.001, tail bias ≥0.001). SNPs identified in positions where the base was uncalled (i.e. ‘n’) in more than one isolate per patient were discarded.

To estimate the number of isolates that need to be analysed to observe all genotypes identified from a patient, random sampling of between one and ten isolates from each set of ten same-patient isolates was performed 100 times for each number of isolates without replacement.

Roary [12] was used to determine gene content variation between isolates from the same patient. Pairs of assemblies were also aligned and compared using the ‘dnadiff’ tool (v1.3), which is part of the MUMmer package [13].

Public Health England holds approvals to process patient-identifiable information for the purposes of infectious disease surveillance, in accordance with Section 60 of the Health and Social Care Act 2001. Ethical approval was not required for this study. The patient specimens were submitted for *Legionella* testing, including culture from microbiology laboratories in England. The *Legionella* data used is collated routinely by the Respiratory and Systemic Bacteria Section, Public Health England (PHE) as part of the national surveillance in England and Wales.

## Results

To investigate the within-host diversity of *L. pneumophila*, we first assessed the diversity of STs (as determined by SBT) amongst each set of ten isolates recovered from ten individual patients with sporadic Legionnaires' disease. In each patient, all ten isolates had the same ST (Table 1).

The number of SNPs amongst same-patient isolates was then determined. The use of a closely related reference genome ensured that maximum resolution was achieved and that almost all SNP-based diversity amongst same-patient isolates would be captured. In seven out of ten patients, no SNPs were detected. In two patients, a maximum of one SNP was observed, and in one patient there was a maximum of two SNPs (Table 1). In each of these three sets in which diversity was observed, nine out of ten isolates were identical, and only one isolate differed by one or two SNPs.

**Fig. 1:**
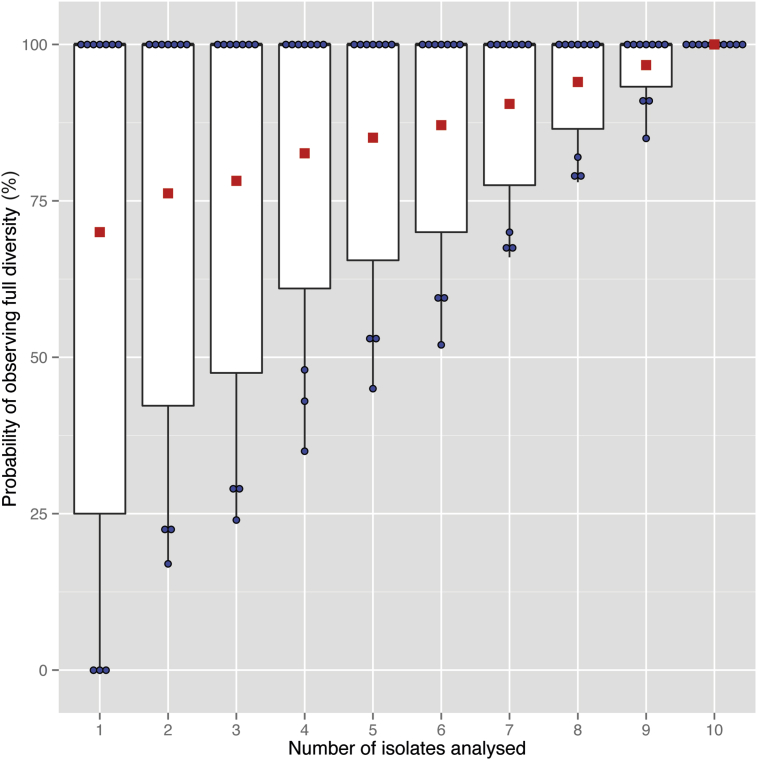
Boxplots showing the probability that all distinct genotypes found amongst each set of ten same-patient isolates are observed when between one and ten isolates are analysed. Probabilities were determined using 100 random samples without replacement for each number of isolates analysed. The blue circles represent the individual probabilities for each of the ten patients, and the red squares show the mean across all ten patients.

Assuming that the full within-host SNP diversity in each set of ten same-patient isolates was captured, we performed random sampling of between one and ten isolates, and calculated the number of times that the full diversity was captured with that number of isolates. Since no SNPs were found amongst isolates from seven out of ten patients, the mean probability of capturing the full diversity with only one sample is 70%. This probability rises as the number of samples analysed increases and, on average, seven isolates are required to have a 90% chance of capturing all genotypes (Fig. 1).

Finally, we investigated the extent of gene content variation between isolates from the same patient. We found no evidence of variation in gene content except for small differences introduced by assembly artefacts.

## Discussion

This study provides the most comprehensive analysis of within-host diversity of *L. pneumophila* in Legionnaires' disease patients to date. The results demonstrate either no or very low within-host diversity in ten patients. We also show that, on average, analysis of one isolate provides a 70% chance of capturing all within-host variation found with ten isolates. Very low within-host diversity has also been observed previously [6], [14], albeit with lower numbers of isolates and patients. Others have reported the opposite, including Coscolla et al. [15] who reported mixed infections in several patients based on SBT profiles from uncultured respiratory samples. Another study that used WGS identified two same-patient isolates belonging to distinct ST191 subtypes that differed by approximately 20 SNPs [16]. However, multiple isolates from three other patients in the same study were identical. Thus, while our study suggests that very low within-host diversity is the norm (at least in sporadic infections), greater diversity has occasionally been observed. Indeed, within-host diversity likely depends on several environmental, clinical and epidemiological factors, including the diversity of *L. pneumophila* in environmental sources, variation in infectious dose between patients, and the duration of infection prior to sampling.

A significant limitation to our study is that the use of culturing procedures may favour growth of some strains over others, thereby reducing the observed diversity. Furthermore, because of the collection of isolates from a single time point, as well as the reliance on culture, it is not possible to determine whether the observed diversity was present at the start of the infection, or whether it evolved during the infection or subsequently in culture. We propose that these limitations may be overcome in future studies by the use of metagenomics on multiple samples obtained over time from the same patient.

## Transparency declaration

JP is a paid consultant to Specific Technologies LLC. VJC is affiliated with, and the research was partly supported by, the National Institute for Health Research Health Protection Research Unit (NIHR HPRU) in Respiratory Infections at Imperial College London in partnership with Public Health England (PHE). This study was funded by Public Health England and the Wellcome Trust (grant number 098051).
